# Investigating the influence of sulphur amendment and temperature on microbial activity in bioremediation of diesel-contaminated soil

**DOI:** 10.1016/j.heliyon.2024.e30235

**Published:** 2024-04-24

**Authors:** Clara Delanau, Thomas Aspray, Mark Pawlett, Frederic Coulon

**Affiliations:** a School of Water, Energy and Environment, Cranfield University, Cranfield, United Kingdom; b Environmental Reclamation Services Ltd, Westerhill Road, Bishopbriggs, Glasgow, G64 2QH, Scotland, United Kingdom

**Keywords:** Soil bioremediation, Respirometry, CO_2_ accumulation, Sulphur amendment, Diesel-contaminated soil

## Abstract

This study investigated the effectiveness of incorporating sulphur (S) with nitrogen (N) and phosphorus (P) for enhancing microbial activity in diesel-contaminated soil during ex-situ bioremediation. While N and P amendments are commonly used to stimulate indigenous microorganisms, the potential benefits of adding S have received less attention. The study found that historically contaminated soil with a moderate concentration of total petroleum hydrocarbons (TPH; 1270 mg/kg) did not have nutrient limitation, and incubation temperature was found to be more critical for enhancing microbial activity. However, soil spiked with an additional 5000 mg/kg of diesel showed increased activity following NP and NPS amendment. Interestingly, NPS amendment at 10 °C resulted in higher microbial activity than at 20 °C, indicating the potential for a tailored nutrient amendment approach to optimize bioremediation in cold conditions. Overall, this study suggests that incorporating S with N and P can enhance microbial activity in diesel-contaminated soil during ex-situ bioremediation. Furthermore, the study highlights the importance of considering incubation temperature in designing a nutrient amendment approach for bioremediation, especially in cold conditions. These findings can guide the design and implementation of future effective bioremediation strategies for petroleum hydrocarbon-contaminated soil.

## 1 Introduction

Ex-situ bioremediation of petroleum hydrocarbon contaminated soils utilises widely distributed indigenous microorganisms capable of breaking down these contaminants [1]. The activity of microorganisms in the soil can be stimulated to enhance contaminant biodegradation. Commonly this involves improving the availability of oxygen and adjusting nutrient concentrations [2]. Nitrogen (N) and phosphorus (P) are often limiting in hydrocarbon contaminated soils, and so these nutrients are applied to soils in their mineral [3] or organic forms (e.g., composts) [4] to stimulate microbial activity.

Concentrations of N and P required to be added for effective remediation have been traditionally calculated by determining the nutrient concentrations within the soil, and adjusted them to achieve an optimal C:N:P ratio (e.g., 100:10:1) for microbial activity [3]. However, this theoretical approach may result in under or over amendment due to an oversimplification of the mixed C present (e.g., mixed biodegradability), as well as, the relative bioavailability of N and P nutrients both present and introduced. Other researchers have similarly demonstrated how optimal C:N:P ratios do not match theoretically references such as 100:10:1 in practice [5]. More recently, N and P limitations have been assessed on a site-specific basis via short-term respirometry assays to determine concentrations that lead to maximal microbial activity measured as CO_2_ production and/or O_2_ consumption [6,7].

There has been relatively little attention on the utility of sulphur (S) for remediation, compared to N and P. S is a key nutrient which can be deficient in soils, for example due to leaching [8]. One relevant laboratory study by Liebeg and Cutright [8], found that a solution containing 75 % S alongside inorganic N and P sources resulted in highest microbial activity in soil historically contaminated with polycyclic aromatics hydrocarbons (PAH). However, in the work by Liebeg and Cutright [8] microbial activity was measured as O_2_ consumption (rather than CO_2_ production) and so the increase in activity with S amendment may not have been associated with contamination degradation/mineralization. Further, as with most laboratory studies, this work considered nutrient amendment at a single temperature. In this case, 30 °C which is well above bioremediation treatment temperatures exposed to in practice in many parts of the world (e.g., polar and temperate regions).

As aforementioned, temperature is another key factor which can influence the performance of ex-situ bioremediation and cold temperatures can decrease microbial activity [9]. In polar and temperate climates, soils in treatment could be actively heated to stimulate microbial activity [10]. In temperate climates, a more cost-effective and sustainable approach would be to schedule bioremediation projects during warmer periods. However, this is often not practicable. Given the importance of both nutrient amendment and temperature on ex-situ bioremediation of hydrocarbons in soil the aims of this research were to ascertain whether; 1) S amendment could stimulate microbial activity (measured as CO_2_ production), and 2) temperature affects the utilisation of S as well as NP. These hypotheses were tested through quantifying the CO_2_ response of microorganisms in diesel-contaminated soil following amendment with inorganic nutrient (NP vs NPS) at two temperatures (10 and 20 °C). The objective of the research was to help maintain and even improve the sustainable delivery of soil bioremediation in temperate climates.

## 2 Materials and methods

### 2.1 Contaminated soil

Historically contaminated sandy gravel soil used in this study was manually sampled from a stockpile of material from a former bus/coach garage site in the Scottish Highlands, UK. Multiple sources of contamination were likely on-site (e.g. fuel oil, mineral oil and lubricating oils) resulting from the site's former use. Due to a lack of information on the specific contamination source for the excavated material, a soil sample was analysed for total petroleum hydrocarbons (TPH) as defined by the TPH criteria working group [11] by gas chromatography flame ionization detection (GC-FID) using an independent confidential UKAS accredited laboratory. The TPH contamination was found to be predominantly composed of compounds with 16–21 carbon molecules (i.e. EC16-EC21 fraction of the TPH-CWG analysis) with a slight dominance of aliphatic over aromatic contamination (Table 1). Based on this analysis, the sandy gravel soil was contaminated with weathered diesel. The TPH concentration of the historically contaminated soil was 1270 mg/kg.

**Table 1:** Total petroleum hydrocarbon contaminated in stockpiled soil.

TPH CWG fraction	Concentration (mg/kg_FW_)
Ailphatic EC5-EC6	<0.001
Ailphatic EC6-EC8	<0.001
Aliphatic EC8-EC10	<0.001
Aliphatic EC10-EC12	11
Aliphatic EC12-EC16	180
Aliphatic EC16-EC21	380
Aliphatic EC21-EC35	110
**Total Aliphatic (EC5-EC35)**	**680**
Aromatic EC5-EC7	<0.001
Aromatic EC7-EC8	<0.001
Aromatic EC8-EC10	<0.001
Aromatic EC10-EC12	3.6
Aromatic EC12-EC16	100
Aromatic EC16-EC21	290
Aromatic EC21-EC35	200
**Total Aromatic (EC5-EC35)**	**590**
**Total TPH**	**1270**

### 2.2 Soil characterisation

The soil samples were air-dried for 24 h to enable them to be sieved through 8 mm, in order to remove stones, plant material and facilitate mixing. Prior to air drying the field moisture content was determined in triplicate by oven drying at 105 °C for 24 h. The standard physical and chemical properties of the soil including pH, moisture content and water holding capacity (WHC) were analysed in triplicate (Table 2). The pH of soil samples was determined using 0.01 M CaCl_2_ (2g fresh weight soil:10 ml CaCl_2_) as previously described [1]. Moisture content was determined gravimetrically after drying at 105 °C overnight. WHC was determined by comparing the moisture content of fresh and water saturated soil. In addition, total organic carbon (TOC), available P (Olsens), available S (extracted using phosphate buffer followed by ICP-OES), nitrate N content (KCl extract and colorimetry) and total N (Dumas method) were determined by independent confidential UKAS accredited laboratories.

**Table 2:** Soil physicochemical characterisation.

Parameter	Unit	Value
pH	n/a	8.2 ± 0.05^a^
Moisture content	%	20 ± 1.1^a^
%WHC	%	29 ± 0.2^a^
Total organic carbon	%	∼3^b^
Available phosphorus	mg/kg soil_DW_	24.7
Available sulphate	mg/kg soil_DW_	76.4
Nitrate Nitrogen	mg/kg soil_DW_	<0.05
Total nitrogen	%	0.161

DW – dry weight; FW – fresh weight; n/a – not applicable; TPH – total petroleum hydrocarbon (sum of TPH-CWG analysis); WHC – water holding capacity.

a n = 3 (standard deviation of values indicated), all other parameters were single measurements.

b Characterisation of sampled soil horizon.

### 2.3 Experimental setup

#### 2.3.1 Historically contaminated soil

Initially, experiments were carried out using historically contaminated soil. Aliquots (50 g dry weight) of historically contaminated soil were added to 250 ml plastic Nalgene® containers. Replicate soils (minimum four per control/treatment) were then amended (2 ml) with either water (referred to as ‘H_2_O control’) or N and P (as NH_4_NO_3_ at 13.30 g/L and K_2_HPO_4_ at 2.6 g/L: referred to as ‘NP’ treatment). The concentration of N used was based on previous research identifying a level of NH_4_NO_3_ suitable across a range of soils [1]. The P concentration was selected to achieve an NP ratio of approximately 10:1. In addition, controls (‘control’) unamended with 2 ml of water were setup in case of moisture change to assess the effect on the microbial activity.

Once prepared, soils were then incubated at 10 and 20 °C for up to 14 days with continual CO_2_ monitoring using the Respicond IV Respirometer.

#### 2.3.2 Spiked soil

In further experiments, the historically contaminated soil described above was spiked with diesel at 5000 mg/kg according to Coulon et al. [12]. As a result of spiking the historically contaminated soil with diesel the hydrocarbon contamination concentration was increased approximately 5 times. The amendment of fresh diesel to the historically contaminated soil was also expected to increase the amount of bioavailable contamination.

A 1 g aliquot of diesel was mixed with 200 g of soil in a glass jar, sealed and left at room temperature for 16 h. Replicate aliquots of diesel and soil were treated in the same way to generate 3 kg of spiked soil. Following this spiking process, aliquots (50 g dry weight) of spiked soil were added to 250 ml plastic Nalgene® containers. Replicate soils (minimum four per control/treatment) were amended with either 2 ml H_2_O (C_H_2_0) or N/P similarly to the ‘unspiked’ soil. Given the higher carbon (contaminant) concentration in the spiked soil, the concentration of NH_4_NO_3_ and K_2_HPO_4_ were increased to 30 g/L and 10 g/L, respectively. In addition, a separate treatment was amended with N, P and S (NH_4_NO_3_ at 30 g/L, K_2_HPO_4_ at 10 g/L and Na_2_SO_4_ at 9 g/L, referred to as the ‘NPS’ treatment) and briefly mixed. The N and P increased from the previous experiment given the increase in TPH present. The concentration of Na_2_SO_4_ used was informed by work of Liebeg and Cutright [8]. In addition, unamended controls (‘C’) were also setup. Once prepared, soils were then incubated at 10 and 20 °C for up to 12 days with continual CO_2_ monitoring using the Respicond IV Respirometer.

### 2.4 Automated respirometry

**Fig. 1:**
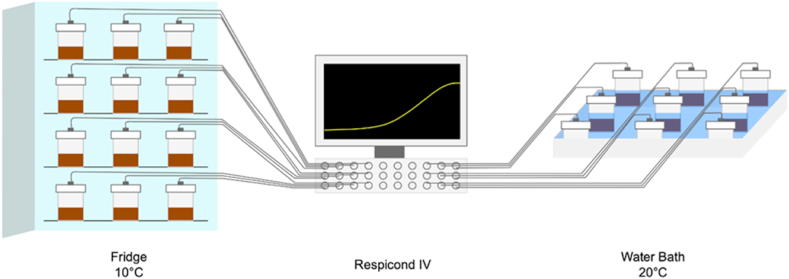
Schematic showing simultaneous incubation of soil samples in a fridge (10 °C and water bath (20 °C) connected to the Respicond IV respirometer.

Nalgene® 250 ml containers with experimental soil samples were connected to a Respicond IV system to measure CO_2_ production over time (Fig. 1). Specifically, CO_2_ produced by soil samples was trapped in 10 ml of 0.6 M KOH solution contained inside each 250 ml container. As CO_2_ was produced by individual soil samples, this was trapped in the KOH solution leading to a change in the conductance of the solution. Two electrodes permanently in the KOH solution of each container (or vessel) were used to measure the conductance controlled by the Respicond system [13,14]. The system was set to measure KOH conductance, and convert this to CO_2_ production, in each vessel at hourly intervals. Given that incubation temperature has an effect on CO_2_ adsorption/measurement with this approach [15], a correction factor was used to compare the results of samples incubated at 10 °C with those at the reference temperature (T) of 20 °C. Specifically, the correction factor (f) applied to the conductivity data was f = [1.014^(20−T)^]. The correction factor was determined experimentally by incubating Nalgene containers without soil at the two temperatures. Our assumption being that the difference in adsorption of residual CO_2_ by KOH traps in the containers at the two temperature was due to incubation temperature only. Therefore, the conductivity at 20 °C was calculated as $C_{20}=fC_{T}$. The accumulation of CO_2_ was then calculated according to the following equation (Eq. 1):

(Eq. 1) $$N\left(CO_{2}\right)=A*\frac{C\left(t_{0}\right)-C\left(t_{1}\right)}{C\left(t_{0}\right)}$$

where C(t_0_) is the conductance at the beginning (time t_0_), C(t_1_) the conductance at time t_1_. *A is* a constant expressing the theoretical maximum amount of CO_2_ absorbed, A = 219 mg CO_2_ for 10 ml of 0.6 M KOH according to Smirnova et al. [16].

### 2.5 Statistical analysis

Accumulated CO_2_ production at the end of incubation (14 days for the “unspiked” and at 12 days for the “spiked” experiments) was analysed by ANOVA. For the “unspiked” experiment treatment variables were Control vs “NP”, and for the “spiked soil” experiment treatment variables were Control, “NP” and “NPS”. Data from the two experiments were analysed separately. Statistical software used was Statistica version 14.0.0.15 (1984–2020) TIBCO. When a significative difference was seen (p < 0.05), data were checked for assumptions and a post hoc test (Tukey-Kramer) test was conducted.

## 3 Results and discussion

### 3.1 Unspiked historically contaminated soil

**Fig. 2:**
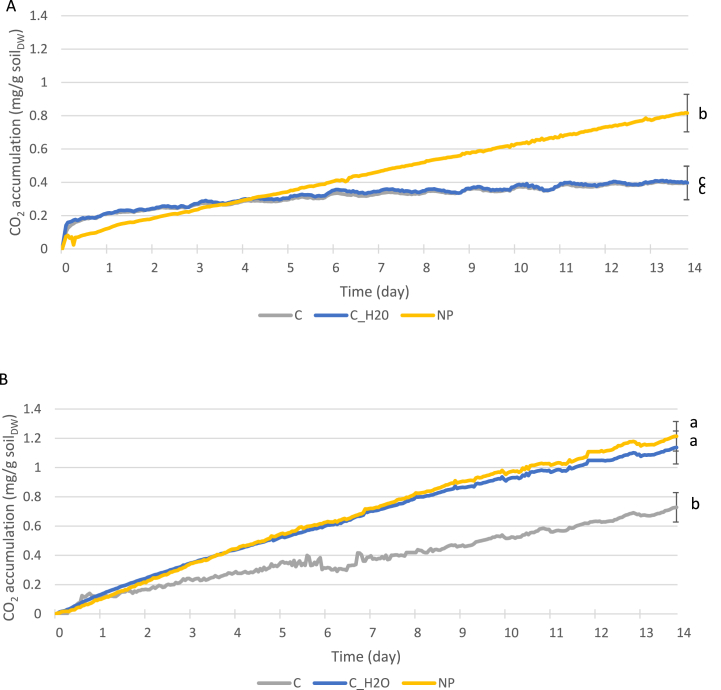
Accumulative CO_2_ production from unspiked soil with time at A) 10 °C and, B) 20 °C. Where, C = control, C_H2O = water amended control, NP = nitrogen and phosphorus amended, NPS the treatment with sulphur in addition to nitrogen and phosphorus. Lowercase letters indicate significant differences between treatments and controls at day 14.

Amendment of the historically contaminated soil with ‘NP’ showed no clear response to the nutrient input at either 10 °C or 20 °C (Fig. 2). A lack of a classical sigmoidal curve response in terms of stimulated microbial CO_2_ production following nutrient amendment suggests that the soil was not limited for NP for the level of TPH contamination present. Hollender et al. [17] found a similar situation in their study with the majority of BTEX and PAH contaminated samples not responding to NP amendment even where available ammonium concentrations were low. These authors assumed that microorganisms were utilising other nitrogen sources in the soil. The results of Hollender et al. [17], and our results here, support the need to use respirometry to determine actual N limitation in hydrocarbon contaminated soils rather than relying on theoretical calculations.

In contrast to nutrient amendment, our results showed a significant (p < 0.05) increase in microbial activity (measured at CO_2_ production) for soil incubated at 20 °C compared to 10 °C (Fig. 2A and B). The increase in activity was independent of nutrient amendment as soil amended with 2 ml of water or NP solution were both more active at 20 °C than 10 °C. Increased microbial activity with increased incubation temperature has been reported in the literature for uncontaminated soil. For example, Pietikäinen et al. [18] showed increased soil respiration in both agricultural and forest humic soils with increased temperature up to 45 °C and 40 °C respectively. These authors found that fungal and bacterial growth rates were optimal at 25–30 °C suggesting an ‘uncoupling’ of soil respiration with microbial (fungal and bacterial) activity at higher temperatures.

Studies on the measurement of soil respiration in hydrocarbon contaminated soils at different incubation temperatures have been lacking till now. By contrast, the effect of different incubation temperatures on hydrocarbon degradation and hydrocarbon-degrading microbial numbers has been studied [19]. Coulon et al. [19] spiked soil with crude oil or diesel to approximately 28,000 mg/kg TPH and found for both contaminants that degradation increased over the temperature range 4–10 °C. Although this supports to a certain degree our results based on soil respiration; Coulon et al. [19] found little increase in TPH degradation between 10 and 20 °C despite hydrocarbon-degrading microbial numbers achieving the highest levels at 20 °C after 3 months of treatment. Coulon et al. [19] found that amendment with N and P also increased TPH degradation.

Given the comparatively low TPH contamination concentration in the unspiked soil studied here, and that the TPH-CWG was indicative of the contamination being weathered diesel, these results have implications on the bioremediation of soils of this nature. Specifically, the results suggest that incubation temperature (which can translate as season in unheated systems) may be more important than N and P amendment in these instances. Therefore, the use of nutrient induced respiration assays such as this support the sustainable delivery of soil bioremediation in respect of nutrient requirements.

### 3.2 Spiked contaminated soil

Given the lack of response to ‘NP’ amendment in the unspiked “historically contaminated soil”, additional experiments were carried out using the same soil spiked with additional diesel to create a situation where NP nutrients were forced to be limiting.

**Fig. 3:**
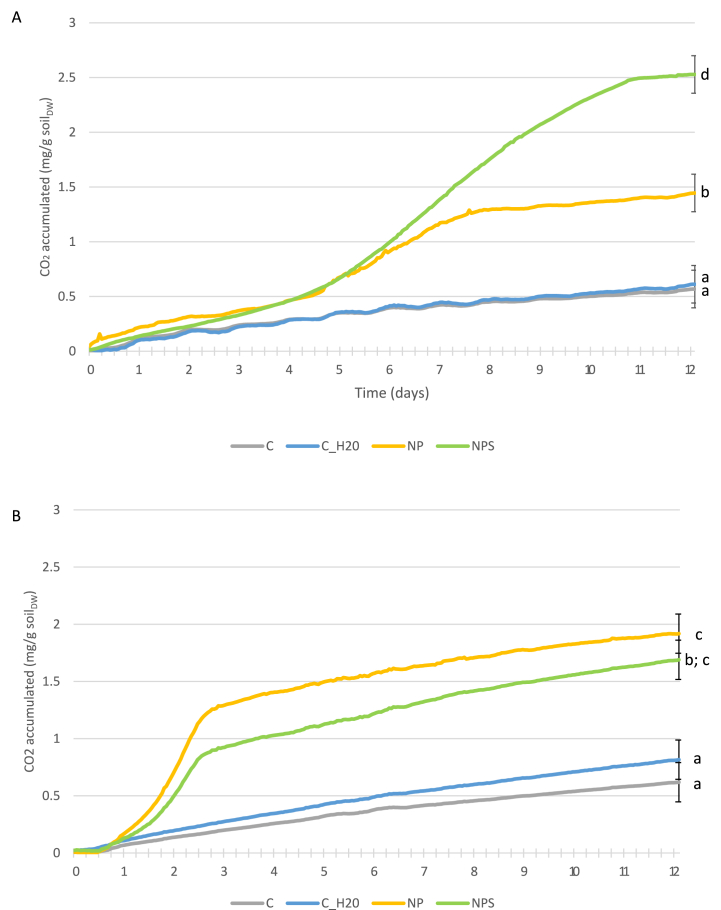
Accumulative CO_2_ production from spiked soil with time at (A) 10 °C and, (B) 20 °C. (C = control, C_H2O = water amended control, NP = nitrogen and phosphorus amended, NPS the treatment with sulphur in addition to nitrogen and phosphorus. Lowercase letters indicate significant differences between treatments and controls at day 12.

Following preliminary experiments with ‘NP’ amendment only, which did show a positive response (data not shown), an experiment was carried out involving amendment of ‘NP’ vs ‘NPS’ amendment (Fig. 3). Specifically, the purpose was to assess the additional benefit of S supplementation on microbial activity (measured as CO_2_ production).

At both 10 and 20 °C, NP amendment increased (p < 0.05) microbial activity above unamended controls (Fig. 3A and B). The curve shape suggests that the lag time was longer in soil incubated at 10 °C, leading to differences (p < 0.05) in accumulated CO_2_ production by day 12. Specifically, the CO2 production for soil incubated at 20 °C and amended with NP being higher than the corresponding treatment at 10 °C.

In contrast to NP, there was a temperature-specific response to NPS amendment (p < 0.05). Soil incubated at 10 °C showing stimulation in microbial activity above NP amended soil. By contract NPS amended soil at 20 °C showed no increase above NP only amendment at this temperature (Fig. 2a and b). As such amendment of S in the form of SO_4_ can enhance microbial activity (under certain conditions) supporting earlier study of Liebeg and Cutright [8] for PAH contaminated soil based on measurement of O_2_ consumption data. A similar result was observed for the same spiked soil amended with a higher concentration of S (data not shown).

The stimulation of microbial activity at 10 °C with NPS amendment suggests that sulphur amendment had a specific impact on psychrophiles (“cold loving” bacteria); considered as microorganisms with a reduced optimum temperature of approximately 15 °C [20]. Even though the spiked soil was adjusted to 85 % soil WHC, which is close to optimal for aerobic microorganisms (60–80 % WHC), it is possible that the stimulated microorganisms were anaerobes. For example, sulphate reducing bacteria utilising the introduced sulphate as electron acceptor (instead of oxygen) for their metabolism. Further respirometry studies using O_2_ consumption alongside CO_2_ production measurement will help elucidate this.

## 4 Conclusion

This study highlights the potential benefits of incorporating sulphur (S) with nitrogen (N) and phosphorus (P) for enhancing microbial activity during ex-situ bioremediation of petroleum hydrocarbon-contaminated soil. This finding complements previous research that focused on amending nitrogen and/or phosphorus. The study also emphasizes the importance of considering micronutrients and trace elements in addition to the key nutrients, N and P, in respirometry assessments to optimize microbial degraders activity. Moreover, the study highlights the importance of temperature in the design of a nutrient amendment approach for bioremediation, especially in cold conditions. This finding has important implications for designing effective bioremediation strategies for hydrocarbon-contaminated soil in polar and temperate regions where psychrophiles and anaerobes may be stimulated by nutrient amendments. Future investigations that incorporate hydrocarbons analysis will further improve our understanding of nutrient supplements that can target contamination mineralization more effectively. Overall, these findings can aid in the development of effective bioremediation strategies for hydrocarbon-contaminated soil.

## Data availability statement

All data available are presented in this study.

## CRediT authorship contribution statement

**Clara Delanau:** Writing – original draft, Methodology, Investigation, Formal analysis. **Thomas Aspray:** Writing – review & editing, Validation, Supervision, Resources, Project administration, Investigation, Funding acquisition, Conceptualization. **Mark Pawlett:** Writing – review & editing, Validation, Supervision, Resources, Project administration, Funding acquisition. **Frederic Coulon:** Writing – review & editing, Supervision, Resources, Project administration, Funding acquisition, Conceptualization.

## Declaration of competing interest

The authors declare the following financial interests/personal relationships which may be considered as potential competing interests:Frederic Coulon is a co-Editor of the Environment Section. Coulon played no part in review and decision making of the acceptance of this manuscript.
